# Vegetables and fruit intake and cancer mortality in the Hiroshima/Nagasaki Life Span Study

**DOI:** 10.1038/sj.bjc.6600775

**Published:** 2003-03-04

**Authors:** C Sauvaget, J Nagano, M Hayashi, E Spencer, Y Shimizu, N Allen

**Affiliations:** 1 Department of Epidemiology, Radiation Effects Research Foundation, 5-2 Hijiyama Park, Minami-ku, Hiroshima 732-0815, Japan; 2 Institute of Health Science, Kyushu University, Kasuga Park 6-1, Kasuga, Fukuoka 816-8580, Japan; 3 Cancer Research UK, Epidemiology Unit, Gibson Building, Radcliffe Infirmary, Oxford OX2 6HE, UK

**Keywords:** cancer mortality, diet, fruit and vegetables, longitudinal study

## Abstract

The association between green-yellow vegetables and fruit consumption and risk of cancer death was investigated in a prospective study of 38 540 men and women who were atomic-bomb survivors in Hiroshima and Nagasaki, Japan. Study participants completed a dietary questionnaire in 1980–1981 and were followed-up for cancer deaths until March 1998, during which time 3136 cancer deaths were identified. Daily or almost daily fruit consumption was associated with a significant 12% reduction in total cancer mortality (RR=0.88; 95% CI, 0.80–0.96 for daily intake compared with intake once per week or less). Daily or almost daily green-yellow vegetables consumption was associated with a marginally significant 8% reduction in total cancer mortality (0.92; 0.94–1.01). Green-yellow vegetables consumption was associated with a significant reduction in liver cancer mortality (0.75; 0.60–0.95). Fruit consumption was associated with a significantly reduced risk of stomach cancer and lung cancer mortality (0.80; 0.65–0.98). Green-yellow vegetables and fruit consumption was associated with a reduction in oesophageal cancer, but these associations were not statistically significant. Neither green-yellow vegetables nor fruit consumption was associated with colorectal cancer or breast cancer mortality. These results support the evidence that daily consumption of fruit and vegetables reduces the risk of total cancer, and specifically cancers of the stomach, liver, and lung.

Since the early 1980s, cancer has been the principal cause of death in Japanese men and women (Health Statistics Foundation, 2000). Although the total cancer mortality rate in Japan is similar to that of other developed countries, mortality rates for specific cancers differ widely. For example, in 2000, the mortality rate for stomach cancer was several-fold higher in Japan (Japan M: 31.2 per 100 000 population; F: 13.8) than in the US (M: 4.5; F: 2.3) or the UK (M: 10.1; F: 4.8); conversely, female breast cancer was up to three-fold lower in Japan than in Western countries (Japan: 7.7; US: 21.2; UK: 26.8) (Ferlay *et al*, 2001). Lifestyle, and in particular diet, has been hypothesised to explain partly the differences in these cancer rates between countries (Tominaga, 1985).

Diet-related factors are thought to account for between 20 and 30% of all cancers (Doll and Peto, 1981). However, few specific dietary factors have been established, except for alcohol intake and cancers of the oral cavity, pharynx, larynx, oesophagus, liver, and breast, and salt intake and cancer of the stomach (World Health Organization, 1977; Smith-Warner *et al*, 1998). Fruit and vegetables have been considered to be as beneficial for several cancer sites, including cancers of the stomach, colon and rectum, lung, pancreas, and bladder, although the epidemiological evidence is inconsistent, particularly for hormone-dependent cancers such as the prostate and breast cancer (Branca *et al*, 2001).

As longitudinal studies of vegetables and fruit and cancer in Japan are scarce (Hirayama, 1986; Kobayashi *et al*, 2002), this study aimed to examine the association between green-yellow vegetables and fruit consumption on the risk of the most common cancer deaths in a prospective cohort study in Japan.

## METHOD

### Study population

The Life Span Study (LSS) is a prospective study of 120 321 subjects including atomic-bomb survivors and nonexposed controls. The Atomic Bomb Casualty Commission (ABCC), followed by the Radiation Effects Research Foundation (RERF), initiated the follow-up in 1950, and regularly monitors the causes of death among the participants through death certificates and other vital status surveys (Pierce *et al*, 1996).

A mail survey was carried out among the 55 650 LSS subjects who were alive as of 1 September 1978 (Radiation Effects Research Foundation, 1978), and of whom 40 349 persons completed the questionnaire (response rate of 72.5%). Completion of the mail survey was effective as from 1 January 1980 for men, and 1 February 1981 for women, during which time 525 persons had died. After exclusion of 1284 cancer cases at baseline based on the information of incidence cases from the Hiroshima and Nagasaki tumour registries, the study population consisted of 38 540 participants (14 873 men and 23 667 women). The mean age at baseline was 56 years old (range 34–103).

### Dietary assessment

The mailed lifestyle questionnaire included questions on past medical history, smoking and drinking habits, marital status, reproductive history, occupation, education, and 22 dietary items, which assessed the average frequency of intake over the previous year (Nagano *et al*, 2000). The validity of the food-frequency questionnaire has been previously reported (Sauvaget *et al*, 2002).

On the food-frequency questionnaire, vegetable intake was limited to green-yellow vegetables, such as pumpkin, carrot, and spinach, and fruit intake referred to total fruit consumption.

For the analyses, green-yellow vegetables and fruit consumption were classified into three frequency categories: ‘once per week or less’, ‘2–4 times per week’, and ‘daily or almost daily’. A subanalysis showed that the mean intake of those with missing data on green-yellow vegetables and fruit consumption was similar to the intake of those who ate these foods once per week or less (Sauvaget *et al*, 2002). We therefore included participants with missing data with those who stated intake of fruit and vegetables as once per week or less (3465 persons with missing information on vegetables frequency intake, and 2257 persons on fruit intake).

### Follow-up/identification of cancer deaths

Follow-up for mortality was linked with the Japanese nationwide family registration system (Koseki). The Koseki provides complete mortality ascertainment for the LSS cohort members residing in Japan. The cause of death was coded according to the International Classification of Diseases, Ninth and Tenth Revisions (ICD-9, ICD-10) (World Health Organization, 1977). The start of the follow-up was assumed to be 1 January 1980 for men and 1 February 1981 for women because the exact dates of receipt of the completed questionnaires were not recorded. The end of the follow-up was defined as the date of death, or 31 March 1998, whichever came first.

### Statistical analysis

The baseline characteristics of the study population were compared according to the intake of green-yellow vegetables and fruit using the χ^2^ test for categorical variables and analysis of variance for continuous variables.

Analyses of the mortality risks were based on multiplicative hazard function models. Relative hazards were computed using the Cox regression methods and confidence intervals were based on the Wald statistics. Age was the primary time scale in all analyses.

Relative risks were estimated for the categories of green-yellow vegetables intake and fruit intake. Baseline rates were adjusted for sex, age (continuous), city (Hiroshima, Nagasaki), radiation dose received by the corresponding organ (continuous), smoking habits (never, current, past), alcohol habits (never, current, past), education level (junior high school or less, high school, junior college or more), and body mass index (BMI=weight/height^2^) (continuous). A test for trend was performed to assess statistical significance across exposure categories by including ordinal terms for each category of intake and entering the variable as a continuous term in the regression model. Lung cancer mortality risks were further stratified by sex and smoking status. For this analysis, current male smokers were dichotomised into those who smoked 20 cigarettes or less per day, and those who smoked more than 20 cigarettes per day. The number of women past-smokers was too small to allow for separate analysis. Relative risks of a specific death as compared to staying alive were calculated using the SAS PHREG procedure (SAS Institute, 2001).

## RESULTS

During a median follow-up time of 16 years, a total of 3136 cancer deaths were identified, which largely comprised cancer of the stomach (*N*=617), liver (*N*=555), lung (*N*=563), and colorectum (*N*=226). On average, women ate green-yellow vegetables and fruit more frequently than men, and never-smokers had a higher consumption of vegetables and fruit than current smokers. Current drinkers ate vegetables and fruit less frequently than never-drinkers, and a high frequency of vegetable and fruit intake was associated with a high education. The mean radiation dose was similar between all categories of vegetable and fruit intake (Table 1

**Table 1:** Baseline characteristics of the study population by tertile of green-yellow vegetables and fruit consumption

	**Green-yellow vegetables**				**Fruit**			
	**Category**				**Category**			
	**1**	**2**	**3**	*P*-value*	**1**	**2**	**3**	*P*-value*
Serving frequency	0–1 week^−1^	2–4 week^−1^	Daily		0–1 week^−1^	2–4 week^−1^	Daily	
Number of subjects	12 397	16 710	9433		9256	12111	17173	
Males sex (%)	46.0	37.2	31.5	0.001	53.1	46.2	25.4	0.001
Hiroshima city (%)	72.8	73.1	74.7	0.002	73.7	71.5	74.6	0.008
Radiation dose mSv (mean)	117.1	112.9	108.1	0.085	116.1	115.7	109.6	0.115
Age (year) (mean)	57.1	54.8	56.8	0.001	57.3	54.7	56.3	0.001
BMI kg m^−2^ (mean)	17.9	20.4	20.2	0.001	17.2	20.2	20.4	0.001
*Smoking habits*								
Never (%)	26.6	45.1	28.3	0.001	16.5	28.3	55.2	0.001
Past (%)	39.8	42.0	18.3		34.1	37.4	28.5	
Current (%)	32.6	42.3	25.1		27.0	33.3	39.7	
*Alcohol habits males*								
Never (%)	37.1	41.2	21.7	0.001	27.8	36.5	35.8	0.001
Past (%)	37.6	43.1	19.4		33.1	39.2	27.7	
Current (%)	39.4	35.9	24.8		33.9	30.7	35.4	
*Alcohol habits females*								
Never (%)	26.5	45.0	28.6	0.001	16.9	27.3	55.8	0.001
Current (%)	29.3	44.9	25.7		19.1	28.2	52.7	
*Education level*								
Low (%)	36.4	42.2	21.4	0.001	28.1	32.8	39.1	0.001
Middle (%)	29.2	45.1	25.8		20.3	30.4	49.3	
High (%)	24.9	42.8	32.3		18.9	32.0	49.1	

* Test for homogeneity of characteristics between categories of green-yellow vegetables and fruit consumption.

*χ*^2^ (sex, smoking, drinking, and education) and analysis of variance.

).

The relative risks for cancer mortality according to the consumption of green-yellow vegetables and fruit for the total cohort are presented in Table 2

**Table 2:** Relative risk according to the level of consumption of green-yellow vegetables and fruit

	**Green-yellow vegetables**							**Fruit**						
		**Category**							**Category**					
	**Total cases**	**1**		**2**		**3**	*P*-value*	**Total cases**	**1**		**2**		**3**	*P*-value*
*Total cancer mortality*														
Cases	3136	1134		1272		730		3136	886		991		1259	
RR (95% CI)		1.00	0.94	(0.86–1.02)	0.92	(0.84–1.01)	0.0676		1.00	0.94	(0.85–1.03)	0.88	(0.80–0.96)	0.0044
*Oesophagus cancer*														
Cases	80	33		31		16		80	30		31		19	
RR (95% CI)		1.00	0.86	(0.52–1.41)	0.89	(0.48–1.63)	0.6348		1.00	0.91	(0.55–1.52)	0.57	(0.31–1.04)	0.0714
*Stomach cancer*														
Cases	617	229		243		145		617	176		203		238	
RR (95% CI)		1.00	0.91	(0.76–1.10)	0.91	(0.74–1.13)	0.3514		1.00	0.97	(0.79–1.19)	0.80	(0.65–0.98)	0.0273
*Liver cancer*														
Cases	555	224		218		113		555	161		165		229	
RR (95% CI)		1.00	0.81	(0.67–0.97)	0.75	(0.60–0.95)	0.0092		1.00	0.85	(0.68–1.06)	0.96	(0.78–1.19)	0.8148
*Gall bladder cancer*														
Cases	157	53		57		47		157	44		38		75	
RR (95% CI)		1.00	0.83	(0.56–1.22)	1.09	(0.73–1.63)	0.7341		1.00	0.69	(0.44–1.07)	0.85	(0.57–1.25)	0.5331
*Pancreas cancer*														
Cases	177	65		74		38		177	49		58		70	
RR (95% CI)		1.00	0.96	(0.68–1.36)	0.82	(0.54–1.24)	0.3641		1.00	1.09	(0.73–1.62)	0.81	(0.55–1.20)	0.2293
*Colorectal cancer*														
Cases	226	80		82		64		226	61		64		101	
RR (95% CI)		1.00	1.04	(0.81–1.35)	1.10	(0.82–1.47)	0.5170		1.00	1.01	(0.75–1.36)	0.97	(0.73–1.29)	0.8141
*Lung cancer*														
Cases	563	214		225		124		563	184		180		199	
RR (95% CI)		1.00	0.98	(0.81–1.18)	0.95	(0.76–1.19)	0.6757		1.00	0.87	(0.71–1.08)	0.80	(0.65–0.98)	0.0348
*Breast cancer (females)*														
Cases	76	15		42		19		76	13		22		41	
RR (95% CI)		1.00	1.64	(0.90–2.98)	1.28	(0.64–2.54)	0.5350		1.00	1.01	(0.50–2.02)	0.91	(0.48–1.72)	0.7006

* Test for trend; relative risk adjusted for sex, age, radiation dose, city, BMI, smoking status, alcohol habits, and education level.

. A daily intake of green-yellow vegetables was associated with a marginally significant 8% reduction in total cancer mortality compared with those who consumed vegetables once per week or less. However, green-yellow vegetables consumption was associated with a significant 25% reduction in liver cancer, with a significant dose–response relationship. Compared with those who ate fruit once per week or less, a daily intake of fruit was significantly associated with a 12% reduction in total cancer mortality, and a 20% reduction in stomach and lung cancer mortality. A daily fruit intake was also associated with a 43% reduced risk of cancer of the oesophagus, but this was not statistically significant. Vegetables and fruit intake was not associated with death from cancer of the colorectum, gall bladder, pancreas, or breast.

Mortality risks were also performed for men and women separately (data not shown). An almost daily consumption of green-yellow vegetables and fruit was associated with a significant 28 and 35% reduction in stomach cancer mortality for women only. And daily fruit consumption was associated with a significant 32% reduced risk of lung cancer in men, but no association was found in women.

Relative hazards for fruit consumption were adjusted for green-yellow vegetables intake and *vice versa*. Furthermore, other food items that were significantly associated with each outcome were also included in the model. However, these adjustments did not materially alter the risk estimates (data not shown). For breast cancer, additional adjustment for age at menarche, age at first birth, parity, breast-feeding, menopausal status, personal history of breast adenoma, and family history of breast cancer also did not materially alter the risk estimates for vegetables and fruit intake (data not shown).

The association between vegetables and fruit intake and risk of lung cancer mortality was stratified by smoking status because smoking may confound the association between vegetables and fruit intake and lung cancer risk (Table 3

**Table 3:** Green-yellow vegetables and fruit consumption and lung cancer risk according to sex and smoking status

	**Green-yellow vegetables**					**Fruit**				
		**Category**					**Category**			
	**Total cases**	**1**	**2**	**3**	*P*-value*	**Total cases**	**1**	**2**	**3**	*P*-value*
**Men**										
*Nonsmokers*										
Cases	15	5	6	4		15	7	5	3	
Relative hazards		1.00	0.90	1.28	0.7517		1.00	0.64	0.19	0.0194
(95% CI)			(0.27–3.07)	(0.33–4.97)				(0.19–2.12)	(0.05–0.79)	
*Past-smokers*										
Cases	47	17	20	10		47	11	16	20	
Relative hazards		1.00	1.38	0.86	0.8142		1.00	1.24	1.06	0.9447
(95% CI)			(0.69–2.78)	(0.38–1.97)				(0.55–2.78)	(0.50–2.26)	
*Current smokers ⩽20 cigarettes day*^−1^										
Cases	189	69	78	42		189	80	62	47	
Relative hazards		1.00	1.06	1.13	0.5385		1.00	0.69	0.67	0.0261
(95% CI)			(0.76–1.48)	(0.76–1.67)				(0.49–0.97)	(0.46–0.98)	
*Current smokers* > *20 cigarettes day*^−1^										
Cases	94	42	35	17		94	34	39	21	
Relative hazards		1.00	0.94	1.06	0.9380		1.00	1.03	0.57	0.0618
(95% CI)			(0.60–1.48)	(0.60–1.87)				(0.65–1.64)	(0.32–1.00)	
										
**Women**										
*Nonsmokers*										
Cases	112	38	46	28		112	19	31	62	
Relative hazards		1.00	0.75	0.68	0.1207		1.00	1.08	0.97	0.8101
(95% CI)			(0.48–1.16)	(0.41–1.12)				(0.61–1.92)	(0.57–1.65)	
*Current smokers*										
Cases	63	23	26	14		63	16	18	29	
Relative hazards		1.00	1.04	1.24	0.5632		1.00	0.99	1.06	0.8304
(95% CI)			(0.59–1.84)	(0.63–2.44)				(0.50–1.99)	(0.56–2.00)	

* Test for trend; relative hazards adjusted for age, radiation dose, city, BMI, smoking status, alcohol habits, and education level.

). Although there were very few lung cancer deaths among nonsmoking men, a daily fruit intake was associated with a significantly reduced risk among both nonsmokers and smokers, whatever the number of cigarettes smoked. In nonsmoking women, a high intake of green-yellow vegetables was also associated with a reduced risk of lung cancer, although this association was not statistically significant, and was not observed among men.

## DISCUSSION

There are limited epidemiological data on the potential protective role of fruit and vegetables intake on cancer risk among Asian populations. This large prospective study, with 18 years of follow-up, found that a daily intake of fruit was associated with a significantly reduced risk of total cancer and a reduced risk of stomach cancer and lung cancer. A daily intake of green-yellow vegetables was associated with a marginally reduced risk of total cancer mortality and a significantly reduced risk of liver cancer mortality.

The potential protective role of vegetables and fruit intake on cancer risk has been extensively studied over the past two decades (World Cancer Research Fund and American Institute for Cancer Research, 1997), and there is consistent evidence that a high intake of fruit and/or vegetables may protect against cancers of the upper gastro-intestinal tract (including stomach cancer). In the present study, vegetables were limited to green-yellow vegetables, defined as vegetables containing more than 1000 IU of carotene per 100 g (carrots, spinach, pumpkin, lettuce, asparagus, parsley, etc.) (Hirayama, 1980). In addition to *β*-carotene, green-yellow vegetables and fruit are a rich source of vitamin C, vitamin E, minerals, and dietary fibres. These nutrients are well known for their immune-enhancing properties and antioxidant effects, especially as *in vivo* markers of lipid peroxidation (Zhang *et al*, 1995; Steinmetz and Potter, 1996; Broekmans *et al*, 2000; Arab *et al*, 2001). They may also act as inhibitors of cancer initiation, promotion, and progression (Hirayama, 1986).

It is well known that high fruit and vegetable intakes are protective factors for upper aero-digestive cancers (Steinmetz and Potter, 1991; Guo *et al*, 1994). Our results are consistent with these findings, although the small number of oesophageal cancers limited the power to detect an association with this cancer site.

Stomach cancer still represents a major cause of cancer mortality in Japan (Health Statistics Foundation, 2000) and in addition to infection with *Helicobacter pylori* (Adam and Efron, 1989), a high salt intake increases risk (Kato *et al*, 1992) and a high intake of fruit and vegetables may reduce risk (Japanese Epidemiological Association on Cancer, 1998). In the present study, the protective effect of fruit and vegetables on stomach cancer was stronger in women than in men. Different exposures between men and women to other stomach cancer risk factors, such as salted or smoked food, smoking, alcohol, and/or stress factors may play a role (Hirayama, 1980; Branca *et al*, 2001), although adjustment for these factors made little difference to results.

There is limited data on the association between fruit and vegetables intake and risk of gall bladder cancer. Our study, based on 157 cases, found no strong evidence for an association between green-yellow vegetables or fruit intake. This is consistent with a previous cohort mortality study conducted in Japan (Hirayama, 1981), although case–control studies have reported significant protective effects for fruit and vegetables (Kato *et al*, 1989) and intakes of vitamins C and E (Zatonski *et al*, 1992). Our study also found no evidence for a strong association between fruit and vegetables consumption and pancreas cancer mortality, which is consistent with other cohort studies (Hirayama, 1981; Zheng *et al*, 1993; Shibata *et al*, 1994; Ziegler *et al*, 1996; Stolzenberg-Solomon *et al*, 2002), although the numbers of cases in all of these studies, including ours, may be too small to detect a moderate association.

Liver cancer and particularly hepatocellular carcinoma is a common cancer in Asia, although rare in North American and Western countries. Until now, the role of diet has been little studied. Our finding that a high intake of green-yellow vegetables significantly decreased the risk of primary liver cancer mortality is consistent with other studies in Japan (Hirayama, 1990), Taiwan (Yu *et al*, 1995), and Italy (La Vecchia *et al*, 1988).

The suggested beneficial effect of vegetables consumption on the risk of colorectal cancer, specifically green leafy vegetables or cruciferous vegetables and their fibre content, has mainly derived from case–control studies (Block *et al*, 1992), but the results of recent large prospective studies have been inconsistent showing either protective effects or no association (Steinmetz *et al*, 1994; Singh and Fraser, 1998; Fuchs *et al*, 1999; Michels *et al*, 2000; Voorrips *et al*, 2000; Terry *et al*, 2001). Indeed, we found no evidence that a high intake of fruit or vegetables is associated with a reduced risk of colorectal cancer death. Further, results from randomised controlled trials have not shown that a diet low in fat and high in fibre and fruit and vegetables can reduce the recurrence of colorectal adenomas (Alberts *et al*, 2000; Bonithon-Kopp *et al*, 2000; Schatzkin *et al*, 2000).

Although breast cancer mortality rates in Japan have steadily increased over the past three decades, the rates are still much lower than that in Western countries (Ferlay *et al*, 2001). It has been suggested that this difference might be partly explained by dietary factors, such as dietary fat or isoflavones (Wynder, 1977; Key *et al*, 1999). The evidence that a high intake of fruit and vegetables is protective against breast cancer is inconsistent (World Cancer Research Fund and American Institute for Cancer Research, 1997), and our findings are in agreement with other cohort studies that suggest that fruit and vegetables intake are not strong dietary determinants of breast cancer mortality in Asian (Hirayama, 1978) or Western women (Verhoeven *et al*, 1997). Other cohort studies have also found no association between vitamin C intake and/or *β*-carotene intake and breast cancer risk (Hunter *et al*, 1993; Rohan *et al*, 1993). However, all these studies included women in middle age or older (40–80 years old) and there is little evidence on the effects of diet at a younger age, when breast carcinogenesis may be initiated (Hill, 1997). The findings from these studies therefore suggest that dietary antioxidants may not have a protective effect at a late stage of breast carcinogenesis. Prospective studies on dietary factors during childhood or adolescence may, therefore, be more appropriate to identify the dietary determinants of breast carcinogenesis.

Lung cancer mortality has dramatically increased in Japan in the last decade and, although it is still below the rates of most developed countries, is now the leading cause of cancer death in men, and the third cause of death in women (Health Statistics Foundation, 2000). Although tobacco smoking is the leading cause of lung cancer, a high consumption of vegetables, fruit, and carotenoids have been associated with a decreased risk of lung cancer (Feskanich *et al*, 2000; Le Marchand *et al*, 2000; Michaud *et al*, 2000; Holick *et al*, 2002). However, randomised controlled trials with supplementation of *β*-carotene and retinol among American male physicians, and male Finnish smokers failed to find any beneficial effect (Hennekens *et al*, 1996; Omenn *et al*, 1996). In our study, the protective effect of fruit on lung cancer mortality was stronger among male smokers compared with past smokers. Although no protective effect was observed in women smokers, a high consumption of green-yellow vegetables tended to decrease the lung cancer mortality in nonsmoking women, of which 70% were because of adenocarcinoma of the lung. This histological type is not related to cigarette smoking but has been associated with hormonal factors and dietary fat (Payne, 2001).

One limitation of this study is that participants might have changed their diet over the relatively long follow-up time. The validation of the questionnaire showed only moderate correlations with the 24-h diary used as reference method (Sauvaget *et al*, 2002). Also, the dietary questionnaire did not include information on portion sizes and was limited to certain foods, so total energy and nutrient intakes could not be calculated. Making no allowance for total energy intake may increase variation in the consumption of specific foods, and attenuate any true association between vegetable and fruit intake and cancer mortality risk. However, it has recently been demonstrated that the correlation between fruit and vegetables consumption and plasma antioxidants was not altered after adjustment for dietary energy intake (Block *et al*, 2001), suggesting that between-person variation in energy intake does not unduly influence fruit and vegetables intake. It should also be noted that by covering only green-yellow vegetables, the questionnaire may have missed an effect of total vegetable consumption on cancer mortality.

On the other hand, the strength of the study lies in the large study population and the long follow-up period. Although the study population is unique in that they are atomic-bomb survivors, radiation exposure was related neither to green-yellow vegetables nor fruit consumption, so the present results may be relevant to other populations.

In conclusion, the present longitudinal study in a Japanese population adds some weight to the hypothesis that a daily consumption of green-yellow vegetables and fruit is associated with a reduced risk of cancer mortality, specifically cancers of the stomach, liver, and lung.
